# Hepatic resection for ovarian cancer in Germany: a nationwide epidemiological study based on DRG (diagnosis-related groups) data (2020–2024)

**DOI:** 10.1186/s12957-025-04064-x

**Published:** 2025-10-20

**Authors:** Miklos Acs, Veronika Müller, Sandra Gebhard, Benedikt J. Wagner, Niklas Bogovic, Markus Götz, Björn Lampe, Hans J. Schlitt, Stefanie Hofmarksrichter

**Affiliations:** 1 Department of Surgery, University Medical Center Regensburg, Regensburg, Germany; 2 Department of Obstetrics and Gynecology, Hospital Mönchengladbach, Mönchengladbach, Germany

## Abstract

**Background:**

Hepatic metastases from epithelial ovarian cancer (EOC) represent an advanced disease stage, yet national guidelines provide no standardized recommendations for liver resection. This study aimed to describe the national trends and patterns of hepatic resections in EOC patients based on German hospital billing data (DRG system) from 2020 to 2024.

**Methods:**

A retrospective epidemiological analysis was conducted using InEK (Institute for Hospital Remuneration Systems) datasets from 2020 to 2024. We extracted data related to diagnosis ICD code C56 (malignant neoplasm of the ovary) and OPS codes for hepatic resections (5-501.0, 5-502.0, 5-502.2, 5-502.3, 5-502.4, 5-502.5, 5-502.6). Case numbers, procedure frequency, hospital characteristics, and DRG classifications were analyzed.

**Results:**

A total of 1,273 hepatic resections were performed in patients with ovarian cancer between 2020 and 2024. Annual case numbers ranged from 225 to 283, indicating stable surgical practice over time. Local excisions / atypical resections accounted for the majority (*n* = 1,165; 91%), while segmentectomies (*n* = 85; 6.7%) and major resections (*n* = 23; 1.8%) were less frequent but consistently performed. Age group analysis showed that the largest proportion of patients undergoing hepatic resection were aged 65–74 years (range 23%–27% yearly). followed by the 60–64 and 55–59 age groups. Younger patients (< 40 years) represented less than 5%. Most procedures (54.6%) were conducted in public hospitals with ≥ 1000 beds, with additional contributions from private non-profit (20.9%) and private for-profit (10.5%) institutions. A gradual increase in the share of private institutions was observed over time. The mean hospital stay varied by resection type: 16.7 days for local excisions, 17.1 days for segmentectomies, and 20.1 days for major resections.

**Conclusion:**

Hepatic resection for ovarian cancer is performed regularly in Germany, primarily at high-volume centers, suggesting a growing clinical acceptance of aggressive cytoreduction strategies despite the absence of formal recommendations. Our findings underline the need for prospective, multicenter studies and guideline updates addressing hepatic metastases in ovarian cancer.

## Background

Epithelial ovarian cancer (EOC) is the most lethal gynecological malignancy, with an estimated 314,000 new cases and 207,000 deaths annually worldwide [1]. In Germany, 7.180 new cases were diagnosed in 2020,rendering it the second most common gynecological cancer entity and the leading cause of death among them [2]. Most women are diagnosed at advanced stages (FIGO IIIC/IV), typically involving extensive peritoneal spread and also affection of upper abdominal organs [3]. Due to the lack of effective screening methods and non-specific clinical symptoms, delayed diagnosis remains a major challenge.

Hepatic metastases occur in up to 26% of EOC cases, either through peritoneal invasion (infiltrative type) or via hematogenous spread [4, 5]. The infiltrative lesions are often addressed as part of standard cytoreductive surgery (CRS), while hematogenous liver metastases have historically been managed more conservatively.

In colorectal cancer, hepatic resection is a guideline-supported and well-established treatment modality [6, 7]. In contrast, the role of liver resection in EOC is less well defined. Current German and international guidelines address hepatic surgery to a marginal extent only and lack concrete recommendations or inclusion criteria [8, 9]. Nevertheless, recent institutional studies have reported encouraging survival outcomes when liver resection is integrated into primary or secondary CRS [10–12].

The aim of this study is to investigate how often liver resections are actually performed in ovarian cancer patients in clinical routine in Germany, and under which circumstances. To answer these questions, we evaluated national hospital billing data (DRG system, InEK((Institute for Hospital Remuneration Systems)) from 2020 to 2024, focusing on procedural patterns, hospital types, and DRG classifications.

## Materials and methods

We performed a retrospective epidemiological study based on hospital billing data from 2020 to 2024 as provided by the German Institute for the Hospital Remuneration System, ‘InEK—Institut für das Entgeltsystem im Krankenhaus’. The InEK system collects data from hospitals through routine documentation of patient treatments. These data include diagnosis codes, procedures and treatment information submitted by hospitals for reimbursement purposes. InEK uses this information to analyze healthcare costs, evaluate resource use and adjust hospital reimbursement rates accordingly.

The analysis was focused on patients with a main diagnosis of ovarian cancer (ICD-10: C56). We extracted procedure data using OPS codes indicating hepatic resections, including:

- 5–501.0.0: Local excision/destruction of liver tissue (atypical liver resection)

- 5−502: Anatomical (typical) liver resection.
- 5−502.0 :Segment resection (one segment).
- 5−502.1: Left hemihepatectomy [resection of segments 2, 3, 4a, and 4b].
- 5−502.2: Right hemihepatectomy [resection of segments 5 to 8].
- 5−502.3: Extended right hemihepatectomy [resection of segments 4 to 8].

Including Trisegmentectomy.

- 5–502.4.4: Bisegmentectomy [left lobectomy] [resection of segments 2 and 3].
- 5–502.5.5 :Resection of other segment combinations.
- 5–502.6.6 :Trisectorectomy [resection of segments 1 and 4 to 8].

The variables included:

- Number of cases per year.
- DRG grouping.
- Hospital characteristics (size and sponsorship).
- Co-registered procedures (peritonectomy, transfusions, bowel resections).

Data were cleaned, aggregated, and visualized using Python.

## Results

Between 2020 and 2024, a total of 1273 hepatic resections were identified in patients with the diagnosis of epithelial ovarian cancer (ICD-10 C56) across German hospitals, based on DRG-coded inpatient procedures. Yearly case numbers were relatively stable, with annual procedures ranging between 225 and 283.

**Fig. 1:**
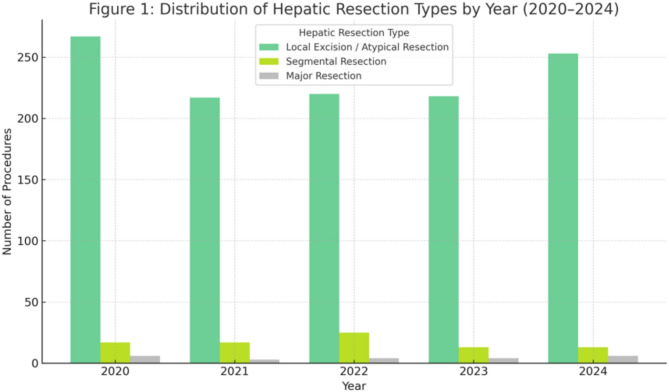
Distribution of hepatic resection types by year

Table 1. and Fig. 1. provide an overview of the total number of hepatic resections per year from 2020 to 2024 across all OPS codes. The distribution of hepatic resection types over time is illustrated in Fig. 1. Local excisions/atypical resections (OPS 5–501.*) were the procedures documented most frequently, while segmentectomies (OPS 5–502.0.0 *) and major hepatectomies (OPS 5–502.1.1–6*) were less common but consistently performed throughout the observation period.

**Table 1:** Annual and total counts of hepatic resection types (2020–2024)

Year	Local Excision/Atypical Resection	Segmental Resection	Major Resection	Total
2020	267	10	6	283
2021	212	13	0	225
2022	217	24	5	246
2023	216	20	5	241
2024	253	18	7	278
All Years Total	**1165**	85	23	1273

### Age distribution of patients undergoing hepatic resection (2020–2024)

**Fig. 2:**
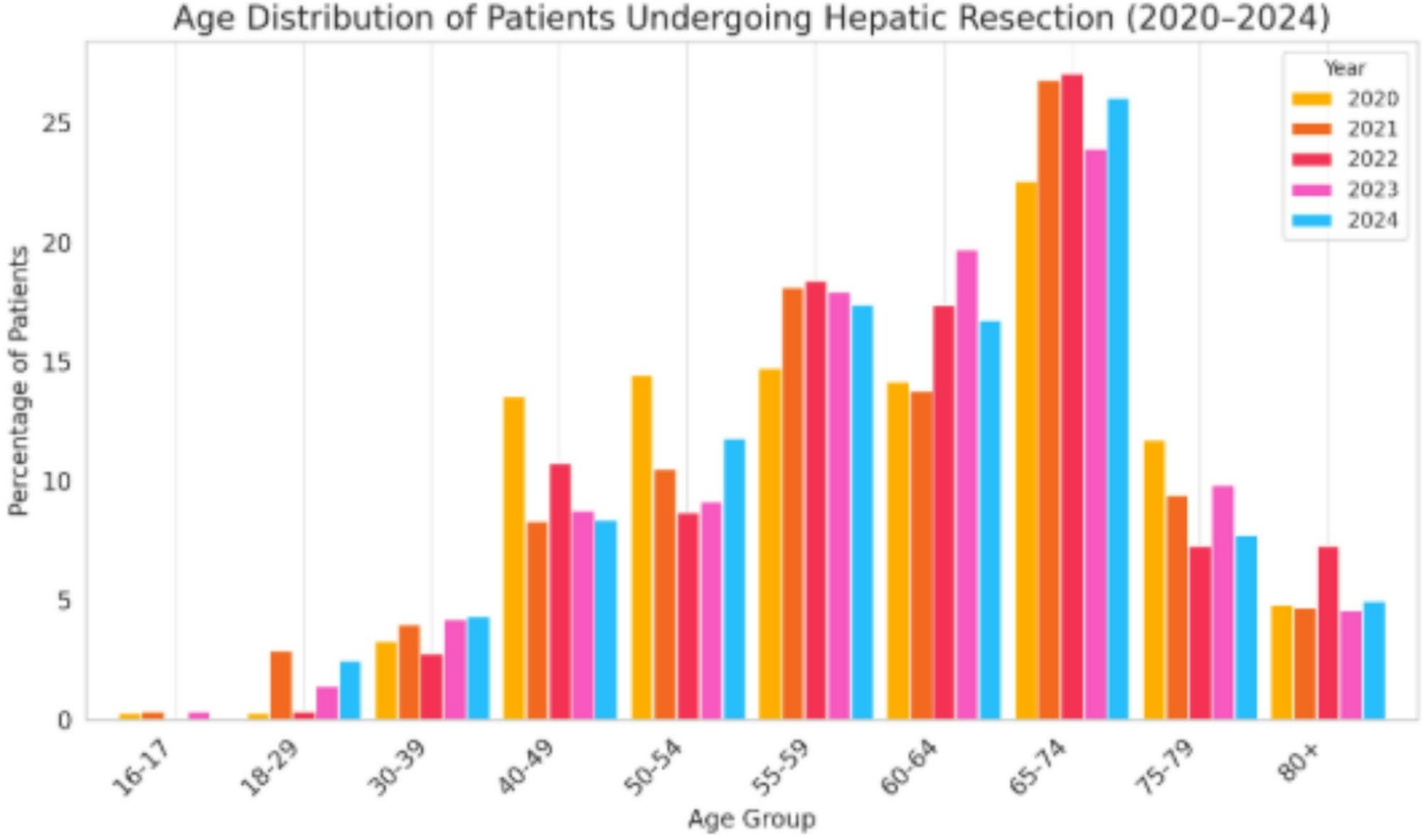
Age distribution of patients undergoing hepatic resection 2020–2024

The age distribution of patients with ovarian cancer who underwent hepatic resection between 2020 and 2024 is shown in Fig. 2. Only age groups aged 16 years and above are included, as no resections were recorded in younger patients across all five years.

The data demonstrate a clear predominance of hepatic resections among older patients. The highest proportion was consistently observed in the 65–74-year age group, accounting for a range from 23% to 27% (mean: 25.30%) of all resections annually. The second-highest frequency was seen in the 60–64 (mean: 16.36%) and 55–59-year cohorts (mean: 17.33%), both showing significant contribution across all years.

Patients aged 16–39 years represented a small minority of cases, with percentages generally below 5%, indicating that hepatic resection is rarely performed in younger individuals with ovarian cancer. The proportion of patients aged ≥ 80 years remained stable, ranging between approximately 4–7% over the study period.

Overall, the age distribution pattern highlights the predominance of hepatic resection in the elderly ovarian cancer population, with the majority of procedures performed in patients between 50 and 74 years of age (Fig. 2).

### Analysis of hospital characteristics

**Fig. 3:**
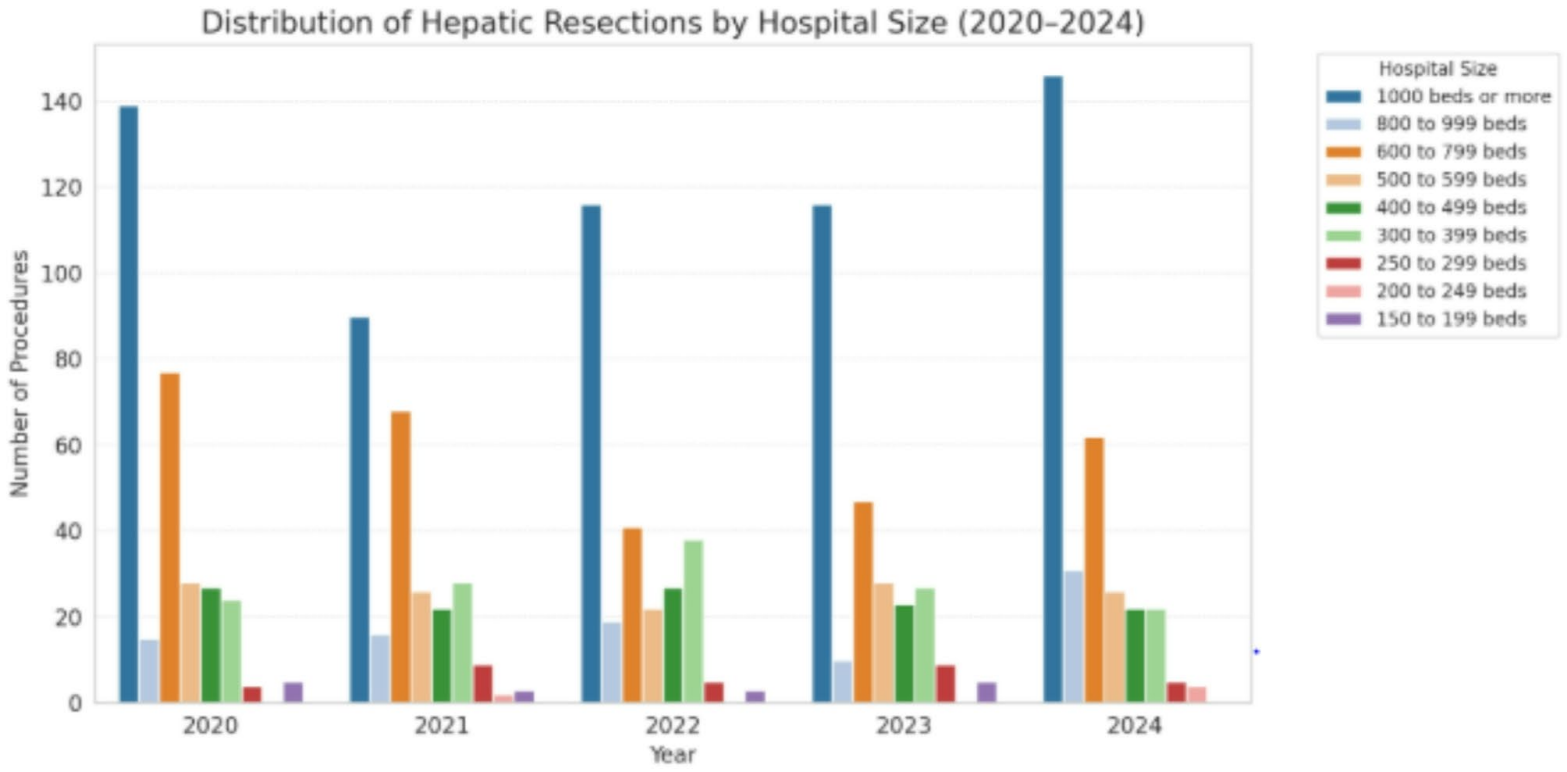
Distribution of hepatic resections by hospital size

Analysis of hospital characteristics revealed that the majority of hepatic resections (54.6%) were performed in large tertiary centers with over 1000 beds. These were predominantly public institutions (62.8%), followed by private non-profit (20.9%) and private for-profit hospitals (10.5%). This distribution suggests that advanced ovarian cancer patients requiring complex surgical care are referred to specialized high-volume centers (Fig. 3).

Regarding reimbursement classification, most liver resections were coded under DRG N01A/B (29.1%), representing extensive gynecological surgery with or without complications. DRG N34Z (20.9%) accounted for resections involving major gastrointestinal or urological procedures in cancer patients. The remaining cases were distributed among DRGs such as G03Z, indicating additional surgical complexity or variation in coding practices across centers.

Procedure combinations commonly included peritonectomy, bowel resection, splenectomy, and intraoperative transfusions. These multimodal approaches reflect aggressive cytoreductive intent. The consistent use of hepatic resection across diverse institutions further underscores its growing clinical relevance, even in the absence of structured recommendations.

### Hospital stay by hepatic resection type (2020–2024) (Fig. 5.)

Based on averaged national DRG data from 2020 to 2024, the mean hospital stay varied depending on the extent of hepatic resection in ovarian cancer patients:

Local excision procedures (OPS 5–501.*) were associated with a mean hospital stay of 16.7 days (range: ~8–25 days).

Segmentectomies (OPS 5–502.0.0) had a slightly higher mean duration of 17.1 days (range: ~10–25 days).

Major hepatectomies (OPS 5–502.1.1, 5–502.2.2, 5–502.3.3, 5–502.4.4, 5–502.5.5, 5–502.6.6) resulted in the longest inpatient care with a mean stay of 20.1 days (range: ~11–29 days).

**Fig. 4:**
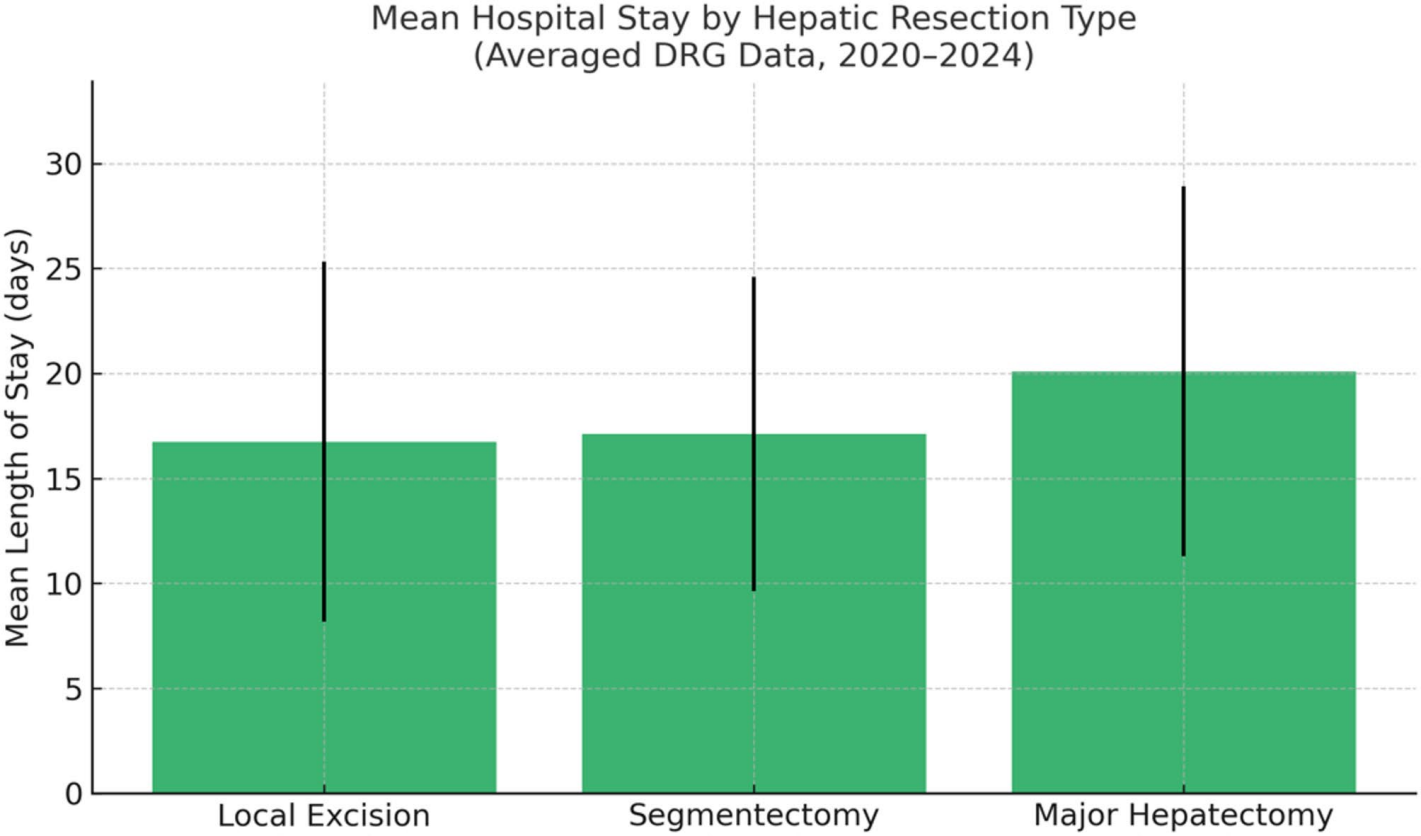
Estimated mean hospital stay (in days) by hepatic resection type in ovarian cancer patients (2020–2024)

These data reflect real-world perioperative resource utilization in tertiary centers and can serve as important references for clinical and economic decision-making in cytoreductive surgery for advanced and recurrent ovarian cancer. These findings are summarized in Fig. 4 and reflect the perioperative burden associated with increasingly complex hepatic procedures.

Peritoneal resections were frequently performed in combination with liver resections, i.e. in 86.3% (*n* = 1099) of the cases. Transfusion was needed in 69.3% (*n* = 882). The high proportion of adhesiolysis (47.4%; *n* = 603) suggests that many patients had prior abdominal surgeries or extensive tumor involvement.

### PCCL = patient clinical complexity level

PCCL is a numeric scale (typically 0 to 6) representing and quantifying the overall severity of comorbidities/complications in hospitalized patients. It is derived from case severity in German hospital data. Hospitals and insurance agencies use PCCL to estimate costs, treatment complexity, and resource requirements.

Table 2. provide the the severity and complexity of a patient’s clinical status from 2020 to 2024.

**Table 2:**
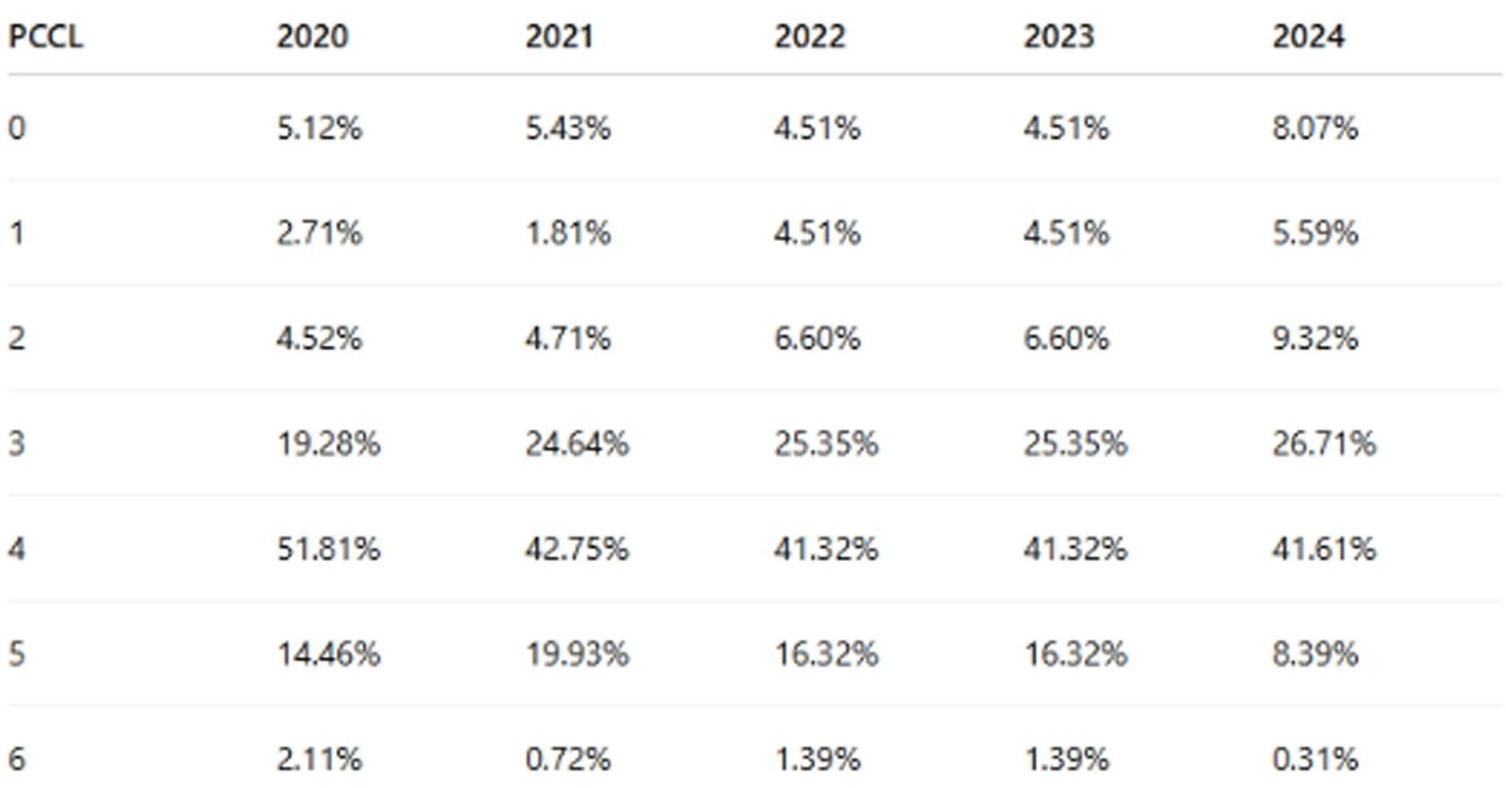
PCCL (patient clinical complexity level) severity distribution from 2020 to 2024

To assess the clinical complexity of patients undergoing liver resections, the Patient Clinical Complexity Level (PCCL) was analyzed for each resection type (Atypical, Segmental, Major) across the study period.

Middle Levels (PCCL 3 and 4) were the most common across all resection types (66–72% of all cases), indicating that most liver resection patients showed moderate to severe complexity. Atypical resections had a slightly higher proportion of PCCL 0–1 cases (14.3%) than segmental resections (11.2%), suggesting they may occasionally be performed in less complex clinical contexts. Major resections, in contrast, were more frequently associated (66.7%) with higher PCCL scores (4–6), reflecting the increased clinical risk and comorbidity burden typically accompanying these more extensive surgical interventions.

**Fig. 5:**
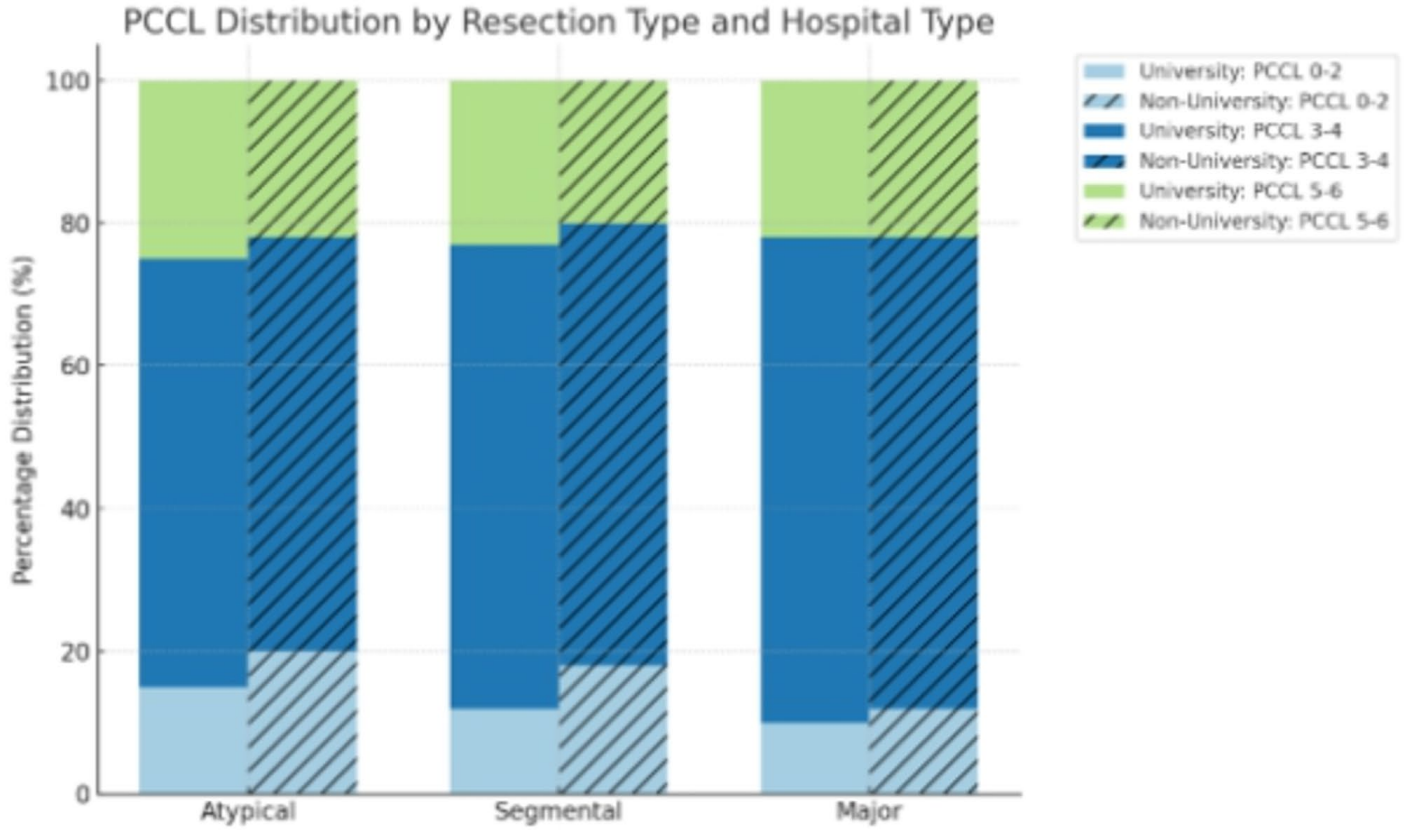
PCCL (patient clinical complexity level) distribution across resection types (atypical, segmental, major) and hospital types (university vs. non-university):

This distribution supports the assumption that atypical resections were often performed for less critical cases. Major resections were more common in clinically complex cases (e.g., multiple comorbidities or severe tumor involvement). Segmental resections fell in between (Fig. 5).

### PCCL by hospital types (university vs. non-university)

From 2020 to 2024 31.9% to 42.2% (mean : 37.6%) of the patients were treated in University hospitals and 57.8% to 68.1% (mean: 62.4%) in non University hospitals.

When stratifying the PCCL distribution by hospital type (university vs. non-university hospitals), the following trends emerged:

Low-complexity cases (PCCL 0–2) were slightly more frequent in non-university hospitals. This pattern was particularly noticeable in atypical resections, indicating either selective patient intake or referral of complex cases elsewhere.

Moderate complexity (PCCL 3–4) remained the dominant category across all resection types and hospital types. The highest shares were observed in segmental and major resections, reflecting the typical clinical burden of liver surgery patients.

High-complexity cases (PCCL 5–6) occurred more frequently in university hospitals(37.6%).This was especially true for major liver resections, suggesting that these centers manage a disproportionately higher share of patients with advanced clinical profiles.

## Discussion

This is the first nationwide epidemiological analysis on hepatic resections in ovarian cancer in Germany. Our data confirm that liver surgery is regularly performed across all federal states, most often in high-volume public hospitals indicating a permanent surgical confidence in performing liver resections as part of cytoreductive strategies, despite the lack of formal guidelines.

The current epidemiological study based on DRG data reveals that 91.5% of liver resections performed in the context of epithelial ovarian cancer (EOC) were classified as atypical or rather local resections. This is in part due to the limitations of the German coding system, in which neither hepatic capsular resections nor partial or complete glissonean capsel resections have a specific procedural code. As a result, it is not possible to differentiate between true parenchymal resections and superficial capsular excisions with minimal or no parenchymal involvement, which likely contributes to the high proportion of procedures coded as 5–501.0.0 (local excision or destruction of liver tissue, i.e., atypical liver resection). Nevertheless, these findings are consistent with existing literature, which indicates that 75% of ovarian cancer cases are diagnosed at an advanced stage (FIGO IIIC/IV) [11]. At these stages, macroscopic peritoneal carcinomatosis extends beyond the pelvis and regularly include infiltration of the hepatic capsule and/or parenchyma, as well as the splenic surface [13]. According to data from the SEER database(Surveillance, Epidemiology, and End Results), liver metastases were present at the time of initial diagnosis in 1,744 patients, accounting for 6.7% of all cases of epithelial ovarian cancer [14]. In recurrent ovarian cancer, the liver is the most common site of solid-organ metastasis within the abdomen, with a reported incidence of 45–48% [15]. Hereby the three major forms of liver involvement in ovarian cancer are [5]: (1) peritoneal dissemination to the hepatic capsule or diaphragm (reported in 40–90% of cases), (2) direct infiltration of the liver parenchyma via peritoneal spread through the capsule (approximately 25% of cases), and (3) true hematogenous liver metastases (in less than 20% of cases). All of these types may be represented within the current dataset; however, due to the lack of detailed operative and pathological information, further differentiation was not feasible. Histopathological confirmation of true parenchymal infiltration was also not consistently available, which represents a key limitation and potential source of bias in our study.

The predominance of atypical liver resections in ovarian cancer patients, highlights the continued preference for limited parenchymal-sparing approaches in hepatic cytoreduction. These local excisions/atypical resections accounted for 1165 out of all 1273 liver resections (91.5%) in the cohort, in contrast to only 85 (6.7%) segmentectomies and 23 (1.8%) major anatomical resections. While atypical resections are associated with shorter hospital stays (mean 16.7 days vs. 20.1 days for major resections), the consistent use of anatomical resections—even in smaller numbers—demonstrates surgical willingness to pursue complete tumor clearance despite increased complexity. This aligns with prior literature showing that achieving no gross residual disease (R0 resection), regardless of anatomical extent, remains the most important prognostic factor in advanced epithelial ovarian cancer [16, 17]. Moreover, studies have shown that anatomical resections may offer superior oncologic control in cases of hematogenous liver metastases due to better margin clearance [12, 18, 19]. Nevertheless, they are also associated with increased morbidity, longer hospitalization, and greater resource demands—factors reflected in our DRG analysis.

The growing role of hepatic surgery in advanced and recurrent EOC reflects the surgical community’s acceptance of aggressive cytoreductive approaches, especially in patients with isolated or limited hepatic disease. Several retrospective studies have supported liver resection as part of secondary or tertiary cytoreduction, showing improved progression-free and overall survival [5, 10, 12]. The consistent use of DRG codes N01A/B and N34Z suggests that hepatic resections are often integrated with major pelvic or intestinal procedures, reflecting a multivisceral strategy. Notably, complex liver resections, such as anatomical segmentectomies and major hepatectomies, were performed in a smaller but substantial subset of patients.

While randomized controlled trials comparing hepatic resection versus chemotherapy alone in ovarian cancer patients with liver metastases are lacking, retrospective cohort data underscore the survival benefit of surgical intervention. In a large NCDB (National Cancer Database) study of stage IV epithelial ovarian cancer patients with distant metastases (including liver), combined chemotherapy and surgery conferred a ~ 50% reduction in risk of death (HR ≈ 0.46–0.49; *p* < 0.0001) compared to chemotherapy alone [10]. Similarly, SEER-based analysis demonstrated that patients undergoing surgery had significantly lower mortality (OS HR ≈ 0.39, CSS HR ≈ 0.37; both *p* < 0.001), and that omission of surgery was associated with much worse outcomes (HR ≈ 4.23) [18]. These findings support the inclusion of surgical cytoreduction—with complete removal of hepatic metastases where feasible—as part of multimodal treatment in selected patients with advanced and recurrent ovarian cancer.

Similarly our group has demonstrated in a retrospective monocentric cohort that complete hepatic resection combined with peritonectomy can be performed safely, even in the context of hyperthermic intraperitoneal chemotherapy (HIPEC), with survival comparable to cytoreduction without hepatic involvement [11]. Other studies [16, 17] support complete resection (no gross residual disease, R0) as the strongest prognostic factor regardless of organ involvement.

Importantly, our data reveal a discrepancy between clinical practice and guideline recommendations. The current German S3 guideline [8] mentions liver resection only peripherally, without defined inclusion criteria. This gap emphasizes the need for updated recommendations based on real-world data and survival outcomes. Furthermore, current epidemiological data show that, despite the lack of guidelines, a consistently high number of patients undergo liver resection in Germany for complete cytoreduction.

Previous reports have shown that the liver is a common site for metastatic spread in ovarian cancer patients with peritoneal carcinomatosis [20, 21] and demonstrated that complete cytoreduction, including hepatic resection, was associated with improved survival. Furthermore, a recent review has supported hepatic resection during primary cytoreductive surgery either in upfront or in interval setting after neoadjuvant chemotherapy [22]. Radiofrequency ablation is also potentially effective alternative to resection [23] which was not included in the current study and represents a competing treatment option for smaller lesions.

In addition to survival benefits, studies indicate that morbidity after liver resection for ovarian cancer remains acceptable when performed in experienced centers [11, 24]. Nevertheless, current data show that incorporating major liver resections into the framework of cytoreduction significantly (*p* < 0.001) increases the mean length of hospital stay compared to local resections (20.1 days versus 16.7 days accordingly). Regarding the extent of resection, this is associated proportionally with corresponding higher morbidity and mortality, but also with additional medical costs. To potentially reduce these factors, the inclusion of hepatobiliary surgeons into gynecologic oncology teams has been highlighted as a key success factor in achieving R0 resection and reducing the complication rate [13].

Given the frequency and institutional concentration of hepatic resections in ovarian cancer patients, our data suggest a growing surgical consensus on the feasibility and relevance of liver surgery in this context. However, the persistent lack of guideline-based indications stands in substantial contrast to the situation in colorectal cancer, where hepatic metastases are clearly addressed in national and international recommendations [7, 25]. We advocate for the development of structured clinical guidelines for hepatic resection in EOC, which would provide a foundation for standardized patient selection, multidisciplinary planning, and outcome benchmarking.

Our findings underscore the differentiated role of hospital types within the healthcare system: university hospitals serve as referral hubs for high-complexity surgical cases, while non-university hospitals manage lower-risk patients more selectively. High-complexity cases (PCCL 5–6) were more frequently observed in university hospitals, which managed an average of 37.6% of patients. This trend was especially pronounced in major liver resections, suggesting that university centers more often treat patients with advanced clinical profiles. Moderate complexity (PCCL 3–4) was the dominant category across all resection types and hospital types, with the highest shares seen in segmental and major resections. This reflects the typical clinical burden in patients undergoing hepatic surgery for ovarian cancer. In addition, our analysis stratified by hospital type (university vs. non-university) revealed significant differences (*p* < 0.05) in patient complexity. Low-complexity cases (PCCL 0–2) were slightly more frequent in non-university hospitals, which treated on average 62.4% of all liver resection patients, particularly evident in atypical resections.

Finally the documentation of liver relapse at the time of secondary resection might be considered as a challenging surgical scenario, thus leading to preclude or limit the achievement of complete eradication of disease. Indeed, several data have been published sustaining that hepatic resection (HR) can be carried out within secondary resection procedures in recurrent ovarian cancer (ROC) patients with acceptable medical and surgical complications. Recently published study has suggested that assessment of mutational status of BRCA could be of help in the decision making approach to surgery versus chemotherapy in platinum sensitive ROC [26]. This study provides an accurate and detailed understanding of the oncological outcomes after surgery for hepatic ROC patients. Furthermore, unique clinicopathological and genetic features were identified that can help predict the prognosis [26]. These findings are highly suggestive of biologic heterogeneity in ROC with liver disease and future studies might reveal genetic signatures associated with better oncological outcomes, possibly opening the way for prognosis stratification, targets of treatment, and a more personalized surgical management. Moreover following extensive image morphological diagnostics the classification should explain the surgical route (laparoscopy vs. laparotomy) of liver resection [27].

Limitations of this study include the lack of detailed clinical parameters such as resection margin (R0/R1), lesion type (infiltrative vs. hematogenous), recurrence status, or adjuvant treatment and long term survival since DRG dataset does not provide data in this regard despite our best efforts. The DRG coding system lacks of further information regarding morbidity types and mortality rates. Furthermore, the aforementioned coding issue complicates the differentiation between true atypical liver resections and procedures involving only superficial excision of the liver capsule. Since both are recorded under the same procedural code (5–501.0.0), it is not possible to accurately distinguish between actual parenchyma-sparing resections and minimal resections limited to the capsule, which may not reflect true parenchymal involvement. These data must be addressed in prospective multicenter trials. Furthermore one major drawback of all registry studies is that the analysis relies on the coding of diseases (ICD-10) and procedures (OPS). Errors in coding, such as misclassification, could not be identified. However, the data provided here contain extensive information about all patients treated for ovarian cancer with liver resection in German hospitals within the specified time frame. Another limitation is that treatment details could not be closely correlated with patient related data, such as comorbidities or ASA (American Society of Anesthesiologists) scores, preventing a thorough risk and outcome analysis.

In conclusion, the national DRG data show a stable pattern of hepatic resection for ovarian cancer in Germany. Our findings encourage guideline bodies to recognize this surgical approach and support the development of clinical algorithms.

## Conclusion

Liver resection represents a consistently utilized and integral component of cytoreductive surgery for ovarian cancer in Germany, with stable case numbers observed over recent years. These procedures are predominantly performed in high-volume tertiary public hospitals and are frequently part of complex multivisceral surgical approaches. The observed patterns point to an emerging national consensus on the role of hepatic surgery in advanced ovarian cancer. These findings underscore the urgent need for harmonized clinical guidelines and prospective studies to evaluate long-term outcomes. Analogous to established recommendations in colorectal cancer, formal integration of hepatic surgery into ovarian cancer treatment guidelines is warranted.

## Data Availability

The data analyzed in this study were obtained from the German Diagnosis-Related Groups (DRG) statistics provided by the Institute for Hospital Remuneration Systems (InEK). Access to these data is restricted and subject to institutional approval and licensing agreements. The datasets are not publicly available due to privacy regulations and data protection policies but may be accessible upon reasonable request and with permission from InEK. Researchers seeking access to the raw data should contact InEK (https://www.g-drg.de) directly and comply with the applicable data use regulations.
